# Bilateral Asymmetry in Ocular Counter-Rolling Reflex Is Associated With Individual Motion Sickness Susceptibility

**DOI:** 10.3389/fneur.2021.759764

**Published:** 2021-11-19

**Authors:** Tomoko Sugawara, Yoshiro Wada, Taeko Ito, Hiroyuki Sakai

**Affiliations:** ^1^Toyota Central Research & Development Laboratories, Inc., Nagakute, Japan; ^2^Department of Otolaryngology, Head and Neck Surgery, Nara Medical University, Nara, Japan; ^3^Wada ENT Clinic, Osaka, Japan

**Keywords:** motion sickness susceptibility, vestibular function, reflex pathway, cortical pathway, ocular counter-rolling, subjective visual vertical

## Abstract

Accumulating evidence suggests that individual variations in vestibular functions are associated with motion sickness (MS) susceptibility. We investigated whether vestibular functions in the reflex and cortical pathways could predict the susceptibility of individuals to MS. MS-susceptible and control adults were recruited according to the Motion Sickness Susceptibility Questionnaire (MSSQ) score. Otolith reflex and cortical functions were assessed using the ocular counter rolling test and the head-tilt subjective visual vertical (HT-SVV) test, respectively. The bilateral asymmetry of each function was compared between the MS-susceptible and the control groups. Although the two tests for otolith functions were conducted using the same stimulation (lateral head tilt), bilateral asymmetry of otolith reflex rather than cortical function was significantly associated with MS susceptibility. Our data suggests that bilateral asymmetry in the otolith reflex pathway is capable of predicting susceptibility to MS to some extent. Our data also suggest that the association between vestibular function and MS susceptibility can vary based on the vehicle types. Future vehicles, such as self-driving cars, will make us aware of other vestibular functions associated with MS susceptibility.

## 1. Introduction

Substantial efforts have been devoted to exploring the determinants of individual motion sickness (MS) susceptibility. Considering the demographic factors, for example, women are more susceptible to MS than men (1–5), age also has a considerable effect on MS susceptibility; young people are more susceptible to MS than elderly people (1, 2, 4, 5). Considering the psychological factors, there is ample evidence indicating anxiety traits to be associated with MS susceptibility (4–6). Identification of individuals who are prone to MS is important for proactive measures such as recommending less provocative transportation or prescribing MS medicine.

Although it is widely accepted that sensory conflicts of motion information between different modalities cause MS (7–9), vestibular function per se is a predominant physiological determinant of MS susceptibility. The most prominent example is of patients with bilateral vestibular loss, who tend to be less susceptible to MS (4, 10). In addition, it is well documented that astronauts with higher asymmetry in bilateral vestibular function were prone to space MS (8, 11–16). These facts suggest that when placed in an altered acceleration environment, asymmetry of bilateral vestibular function, especially otolith functions, which rarely manifests in daily life can play a role in the development of MS.

However, it remains unclear which functions in the vestibular pathway are associated with MS susceptibility. Vestibular signals from the end organs (otoliths and canals) reach the vestibular nuclei *via* the vestibular nerve (17–19). The signal is then divided into two functionally distinct pathways, one of which is the reflex pathway, which plays a critical role in stabilizing gaze and posture (20). The other is the cortical pathway *via* the thalamus (21–23), which is critical for spatial orientation (21, 24, 25). Considering the vestibular reflex pathway, there are few studies suggesting that reflex sensitivity is associated with MS susceptibility (26–28). In contrast, to the best of our knowledge, no data exist on the relationship between vestibular cortical functioning and MS susceptibility in healthy individuals. The best way of filling this gap in the literature is to examine vestibular functions in both the reflex and cortical pathways in the same study population in relation to MS susceptibility.

In the current study, we aimed to investigate whether vestibular functions in the reflex and cortical pathways could predict individual MS susceptibility. Specifically, vestibular functions in the reflex and cortex pathway were assessed using two clinical tests: the ocular counter-rolling (OCR) test for otolith-reflex sensitivity to detect linear acceleration (13, 14, 29, 30) and the head-tilt subjective visual vertical (HT-SVV) test (31, 33). There are a variety of tests that assess the bilateral asymmetry of otolith function. Among these, a widely used clinical test is the vestibular evoked myogenic potentials (VEMP) test in which each unilateral otolith can be separately stimulated by bone/air conduction of sound (34, 35). The VEMP and OCR are complementary tests that evaluate different aspects of otolith function (36). For the aim of this study, we adopted the OCR and HT-SVV tests providing different outcomes to compared the impacts of asymmetry in reflex and cortical pathways on MS susceptibility using the same vestibular stimulation (i.e., lateral head tilt) with the HT-SVV test. Meanwhile, the OCR test produces a different outcome, torsional eye movement around the gaze axis, from the HT-SVV test that measures subjective vertical tests bring different outcomes, that is, torsional eye movement around gaze axis and subjective vertical, respectively. Finally, bilateral asymmetry in each vestibular sensitivity was compared between MS-susceptible and control adults.

## 2. Materials and Methods

### 2.1. Participants

**Figure 1:**
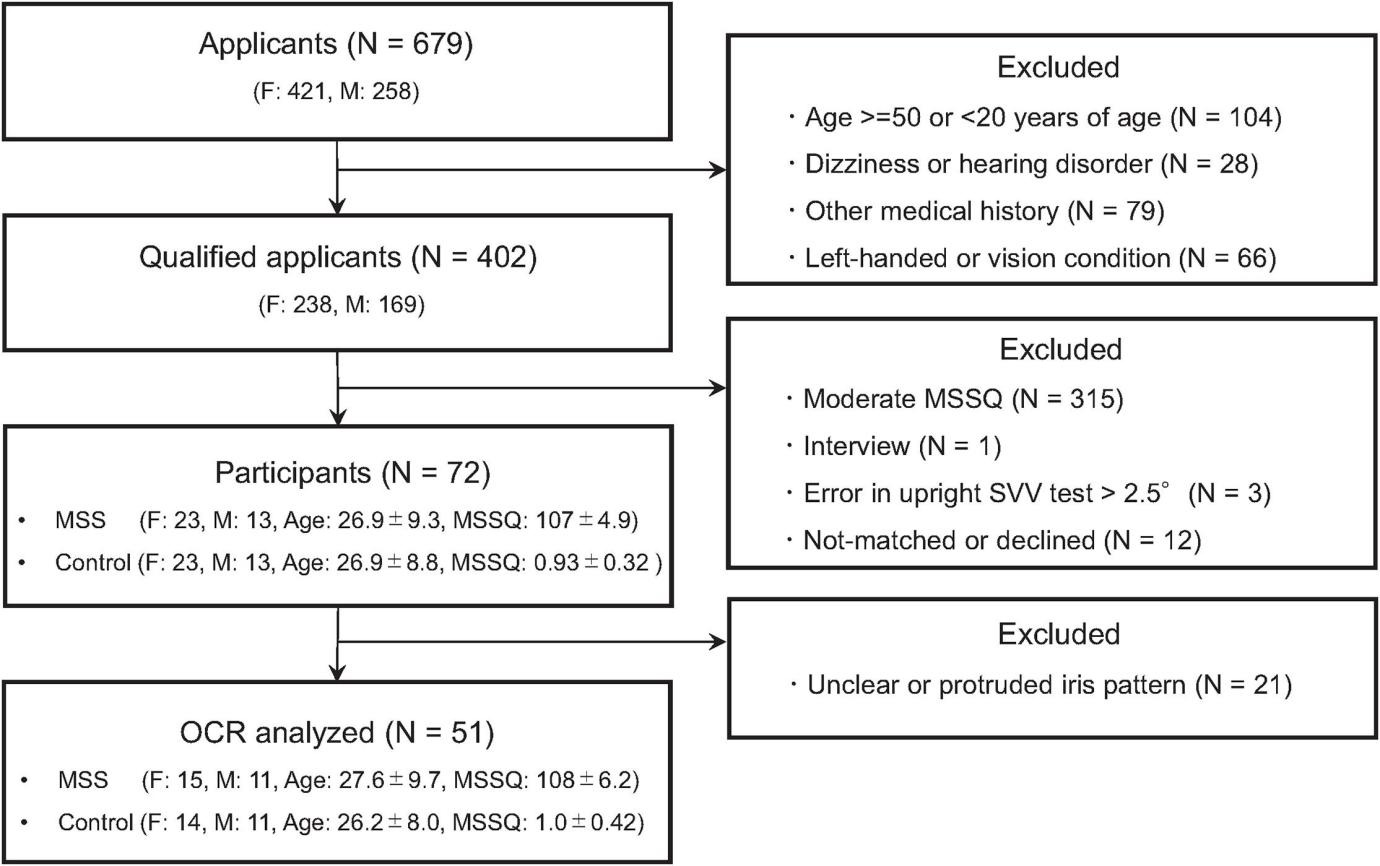
Flowchart of the screening process. N, F, and M denote numbers of group, female and male participants, respectively. Age and MSSQ denote means ± SEM of age and Motion Sickness Susceptibility Questionnaire (MSSQ) score, respectively.

Healthy adults aged between 20 and 50 years were recruited through an employment website. Among the 679 applicants, 277 were excluded for various reasons (details in Figure 1). The remaining 402 applicants were further screened using the Motion Sickness Susceptibility Questionnaire (MSSQ) (37, 38) and clinical tests by otolaryngologists (TI and YW). The MSSQ assesses the experience of typical MS symptoms (e.g., nausea and vomiting) for each of the nine different vehicles (e.g., cars, buses, and ships) in childhood (before 12 years of age) and the last 10 years. The MSSQ score was calculated as the total of the symptom rating scale by the number of vehicle types causing MS in the individual. Applicants with moderate MSSQ scores (25–75th percentile) were excluded to ensure that participants with extremely different susceptibilities to MS were enrolled. The clinical tests consisted of diagnostic interviews, the body sway test evaluating balancing function (39), the video head impulse test (vHIT) evaluating canal sensitivity (40–42), and the head-upright SVV test evaluating vertical sensitivity (31). These tests were performed to ensure that participants had normal vestibular function (refer to Supplementary Material for more details). Consequently, four more applicants were excluded. In total, 36 individuals highly susceptible to MS (MS-susceptible) and their age- (within ±3 years) and sex-matched controls less susceptible to MS (controls) participated in the OCR and the HT-SVV tests. We also acquired functional and structural neuroimaging data using magnetic resonance imaging to investigate the involvement of brain functions in MS susceptibility. TThese results has been reported elsewhere (32). Written informed consent was obtained from all the participants after an in-person explanation. The protocol and consent form were approved by the institutional review board of Toyota Central R&D Labs., Inc. (IRB number 16-09, 2016) and approved by the Ethics Committee of Nara Medical University (IRB number 916, 2014). The study was performed in accordance with the Declaration of Helsinki.

### 2.2. HT-SVV Test

The HT-SVV test was conducted by experimenters following the steps delineated by Wada et al. (33) using a computer-aided measurement system (UniMec, Tokyo, Japan). Participants sat on a stool in a dimmed room facing a visual display device that presented a luminous bar. Goggles with the built-in accelerometer (KXR 94-2050, Kionix in UniMec SVV measuring system) were worn to pick up gravitational acceleration and to occlude any visual cues except for the bar. When the test was initiated, participants were asked to close their eyes and keep their head in a certain position (upright, 30° left tilt, or 30° right tilt in a pseudo-random order). The experimenter constantly monitored head-tilt angle provided by the measuring system during the test procedure. After the experimenter randomly tilted the bar either clockwise or counter-clockwise, participants opened their eyes and were provided with sufficient time to return the bar to the subjective vertical position using a manual controller. This procedure was repeated six times for each head position. The system recorded both the alignment errors of the bar and mean head-tilt angles with respect to gravitational vertical and computed the left and right otolith sensitivities, *S*_*l*_ and *S*_*r*_, respectively. Thus, we calculated the HT-SVV asymmetry index, *I*_*SVV*_ by the following equation:

(1) $$\begin{array}{r} I_{SVV}=|S_{l}-S_{r}|/\left(S_{l}+S_{r}\right) \end{array}$$

### 2.3. OCR Test

The OCR test was followed by the vHIT and was performed by the otolaryngologist using a video oculography device (EyeSeeCam, EyeSeeTec GmbH, Germany). Participants were asked to fixate on a visual cue throughout the test. The experimenter first held the head of the participant in an upright position for 20 s, and then tilted the head by 30° to the left or right, held the head in the tilted position for 20 s, and finally returned the head to the original upright position. The same procedure was repeated two or more times. Head-tilt angle was calculated based on the gravitational acceleration monitored by a built-in accelerometer of the device. The experimenter constantly monitored the head-tilt angle provided by the measuring system during the entire test procedure, while the measuring system recorded the left eye image of the participant and head-tilt angle with a sampling rate of 10 Hz. After the test, we estimated torsional eye movements by applying an iris template-matching algorithm to the recorded eye images. In short, a partial iris pattern of an eye was extracted from a single video frame at the upright head position and stored as a reference pattern. Then, eye torsional angle was determined by searching for the best matching position of the reference pattern in the iris images from the video frames other than the reference frame (refer to Supplementary Material for more details). Thus, we calculated the left and right OCR gains, *O*_*l*_ and *O*_*r*_, respectively, by dividing the estimated torsional angle by the head-tilt angle, and then determined the OCR asymmetry index, *I*_*OCR*_, as follows:

(2) $$\begin{array}{r} I_{OCR}=|O_{l}-O_{r}|/\left(O_{l}+O_{r}\right) \end{array}$$

### 2.4. Statistics

Welch's *t*-test was used to clarify the difference in asymmetry index between the MS-susceptible and control groups using a statistical tool, R studio (ver. 1.1.456, R Core Team, 2018). For reporting effect size, a statistical tool G*Power 3.1.9.7 was used. The correlation between the asymmetry indices was tested for significance. In all the calculations, a significance level of 0.05 was used.

## 3. Results

**Figure 2:**
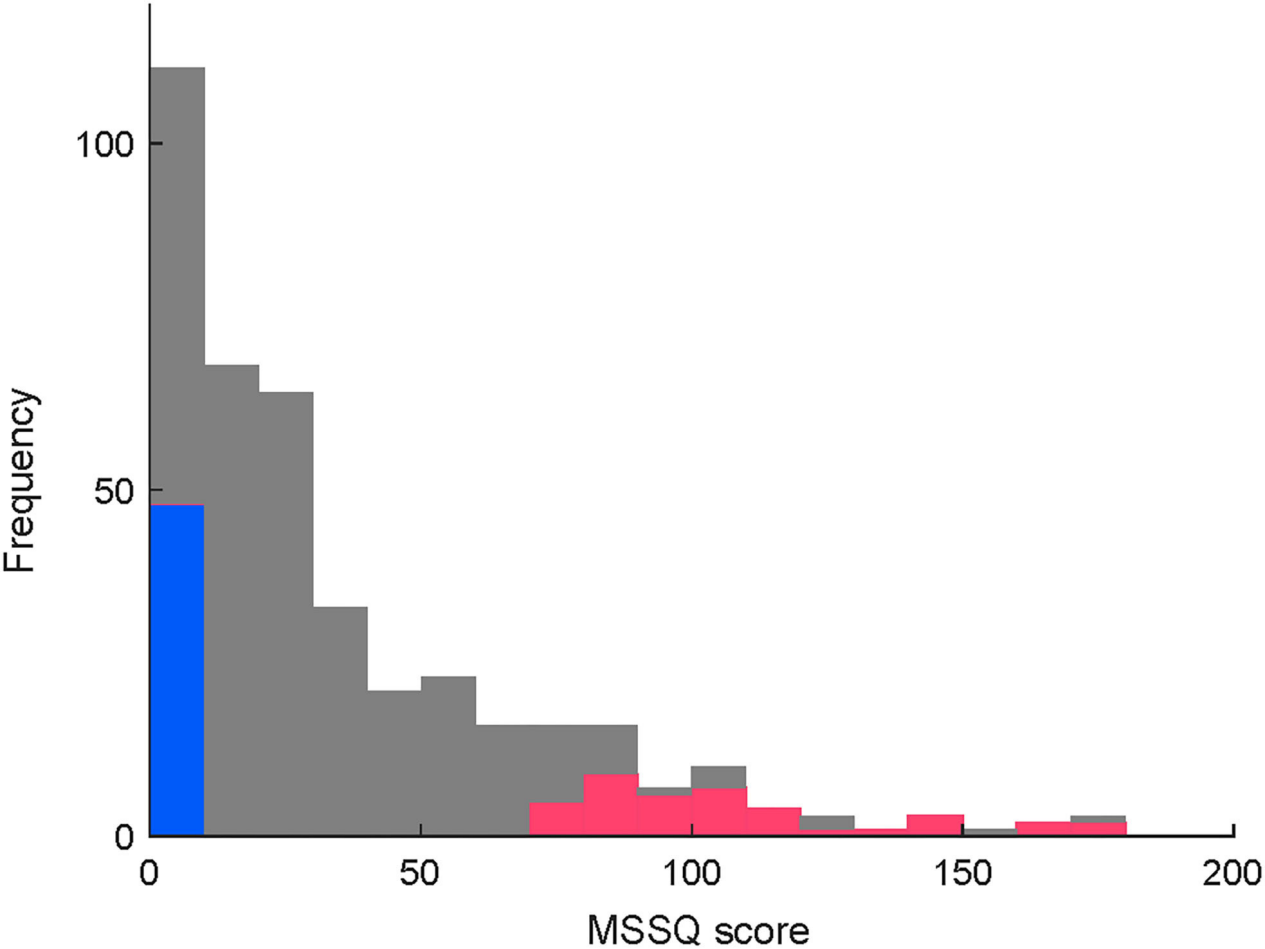
(Caption of Y is revised) Distribution of MSSQ scores in the qualified applicants. Colored parts of both ends depict MSS (red, < 25th percentile) and control (blue, > 75th percentile) participants, respectively.

Figure 2 is a histogram of the MSSQ scores. Among 402 qualified applicants, MS-susceptible (*n* = 36) and control (*n* = 36) groups were selected from both ends of the histogram. All the participants completed the vestibular function tests and were endured to have clinically normal vestibular function. More importantly, there were no between-group differences in the body sway test or the vHIT test (*P* > 0.1, Supplementary Material for more details). Meanwhile, adequate image quality for the OCR test was not acquired in 21 participants. Thus, the statistical tests of between-group differences in asymmetry indices were conducted in a subpopulation of the participants consisting of 26 MS-susceptible and 25 controls (Figure 1).

**Figure 3:**
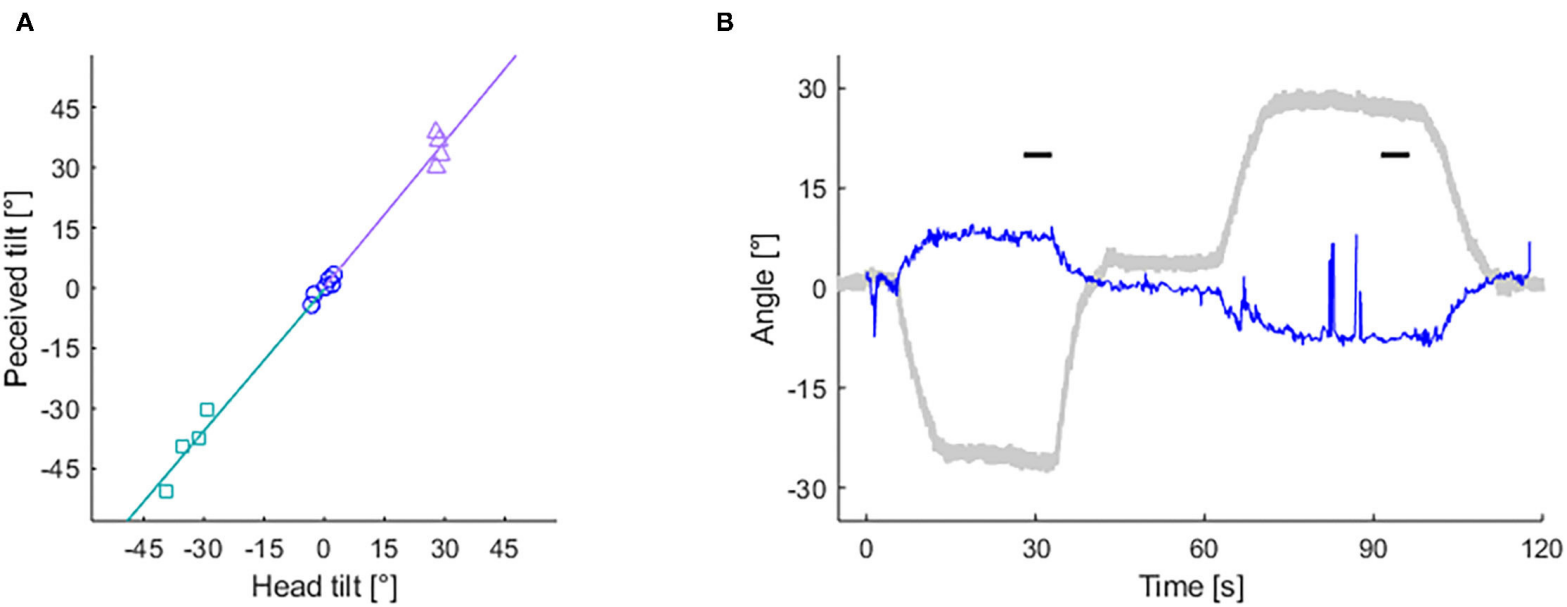
Records of subjective visual vertical (SVV) and ocular counter-rolling (OCR) tests from a MS susceptible individual. **(A)** Slope of lines depict left (based on rectangle-dots) and right (based on triangle-dots) head-tilt subjective visual vertical (HT-SVV) gain (*S*_*l*_ and *S*_*r*_, respectively). Circle dots are used to calculate head-upright SVV. **(B)** Eye torsion (blue thin line) and head tilt (gray bold line) angle during the OCR test. Short horizontal bars depict 5 s time periods used for calculating OCR gains.

Figure 3 shows example results of the HT-SVV test and the OCR test from a MS-susceptible individual (49 year-old female). In this individual, gains and asymmery indices were calculated as follows: *S*_*l*_ = 1.18, *S*_*r*_ = 1.21, *I*_*SVV*_ = 0.013, *O*_*l*_ = −0.25, *O*_*r*_ = −0.35, *I*_*OCR*_ = 0.17.

**Figure 4:**
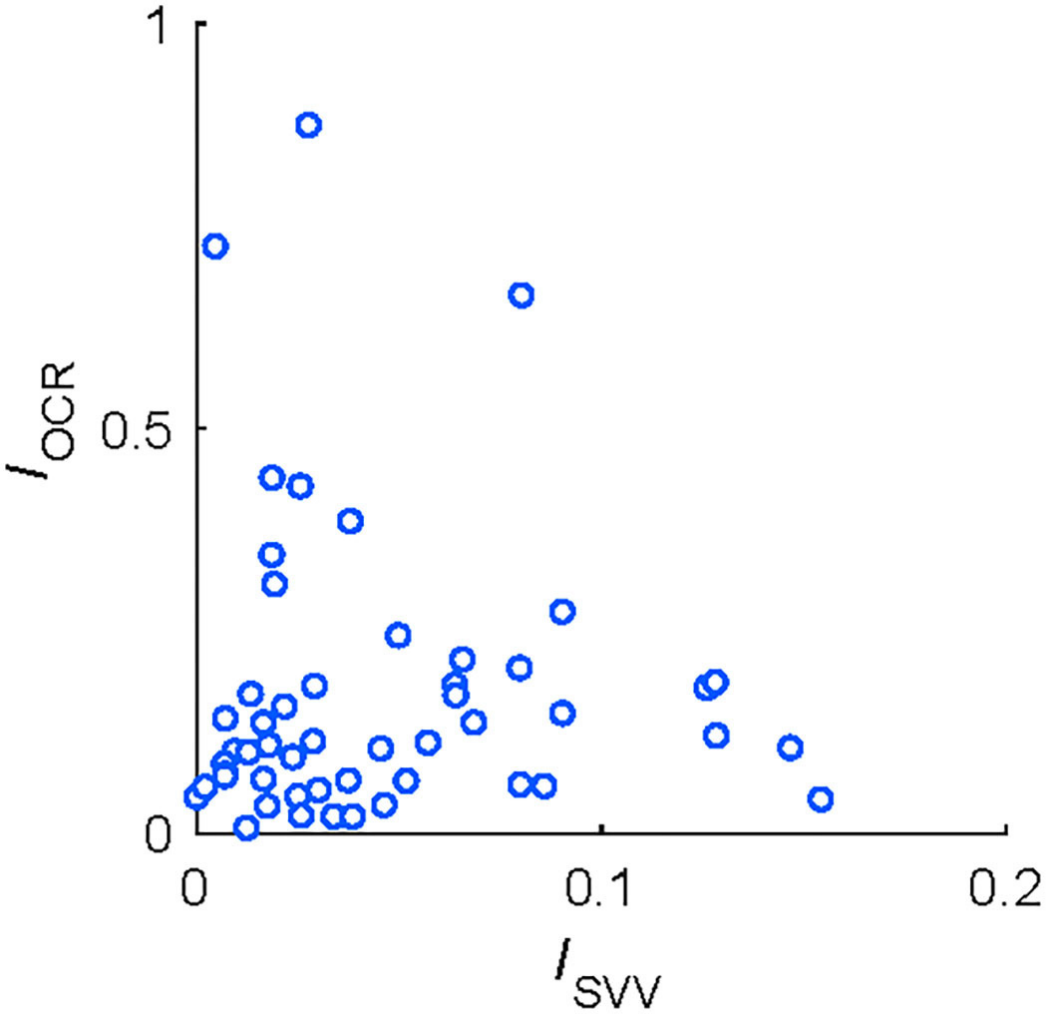
Correlations between functional asymmetry in HT-SVV (*I*_*SVV*_) and in OCR (*I*_*OCR*_) tests from 51 participants.

Figure 4 shows the pairwise correlations between asymmetry indices of vestibular functions. There were no significant correlations between OCR asymmetry and HT-SVV asymmetry (*r* = −0.03, *P* = 0.83; (Figure 4), indicating that each index could assess an independent aspect of vestibular functional asymmetry.

**Figure 5:**
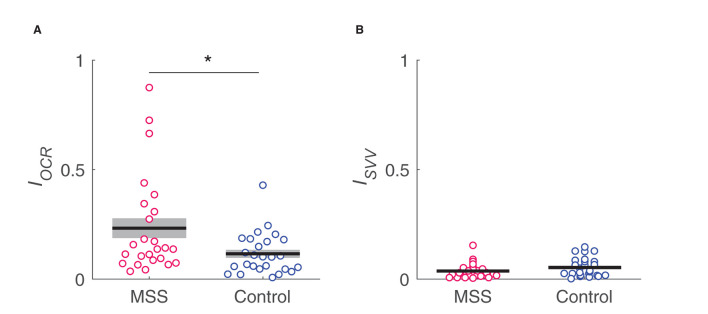
Comparison in IOCR **(A)** and Isvv **(B)** asymmetry indices, respectively, between MS susceptible (MSS) and control groups. *indicates a significant difference.

Table 1 summarizes the results of the OCR and HT-SVV tests. Between-group comparisons revealed a significantly greater asymmetry index of OCR gains in the MS-susceptible rather than the control group Figure 5A) and the comparable asymmetry index of HT-SVV gains between the two groups Figure 5B). In Figure 5A, some outliers were apparent. Thus, we performed Smirnov-Grubbs' outlier test in each group and found three from the MS susceptible and one from the control group as outliers. Having excluded these four outliers, the significant difference remained between the two groups after Welch's *t*-test (*d* = 0.66, *P* = 0.031). Although similar vestibular stimulation (lateral head tilt) was used in both the OCR and the HT-SVV tests, OCR asymmetry was significantly greater than HT-SVV asymmetry (MS-susceptible: *d* = 0.84, *P* < 0.001; control: *d* = 0.65, *P* = 0.003; paired *t*-test).

**Table 1:** Mean and SEM of vestibular function tests in motion sickness susceptible (MSS) and control groups.

	**MSS (*n* = 26)**	**Control (*n* = 25)**	**Effect size g**	**P-value**
*O*_*l*_	0.209 ± 0.019	0.201 ± 0.014	0.09	0.74
*O*_*r*_	0.237 ± 0.021	0.207 ± 0.016	0.31	0.27
*I*_*OCR*_	0.230 ± 0.044	0.113 ± 0.019	0.68	0.019^*^
*S*_*l*_	1.07 ± 0.018	1.07 ± 0.024	0.01	0.97
*S*_*r*_	1.11 ± 0.029	1.13 ± 0.033	0.08	0.77
*I*_*SVV*_	0.038 ± 0.007	0.052 ± 0.009	0.37	0.19

* *indicates a significant difference*.

To further confirm the non-significance of HT-SVV asymmetry with a larger sample size, we performed between-group comparisons using all the participants who completed the test (i.e., *n* = 36 for MS-susceptible group and *n* = 36 for control group). Consequently, the result was replicated in HT-SVV asymmetry (MS-susceptible: 0.046 ± 0.007; control: 0.044 ± 0.007; *g* = 0.03, *P* = 0.90).

**Figure 6:**
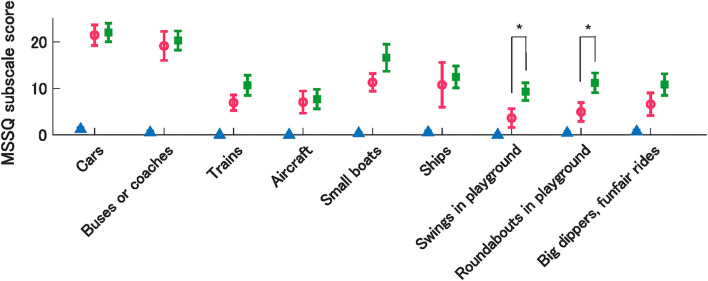
Mean MSSQ sub-scores by vehicle types from the control group (triangle) and MSS subgroups with high (circle) and low (rectangle) OCR asymmetry. Error bars denote SEM. *indicate significant differences between MSS subgroups.

Moreover, we compared the MSSQ subscale scores for each vehicle type between individuals with low and high OCR asymmetry in the MS-susceptible group. Using the mean OCR asymmetry index as a threshold, the MS-susceptible group was further divided into low (*n* = 18) and high (*n* = 8) asymmetry subgroups. Between-group comparisons in the MSSQ subscale scores revealed, interestingly, that the low OCR asymmetry group was more susceptible to motion stimuli in playground equipment, such as swings and roundabouts, compared to the high OCR asymmetry group. These differences were statistically significant after Bonferroni correction for multiple comparisons (*P* = 0.001, *g* = 1.12 for swings; *P* = 0.002, *g* = 1.03 for roundabouts; Figure 6).

## 4. Discussion

In the current study, we examined which vestibular functions were associated with MS susceptibility. The results showed a significant association of MS susceptibility with OCR, but not HT-SVV asymmetry. In addition, neither postural control (body sway test) nor semicircular canal function (vHIT) was associated with MS susceptibility. Taken together, these findings suggest that functional asymmetry in bilateral utricles perceiving lateral head acceleration in the vestibular reflex pathway may play a role in individual susceptibility to MS, and this role is not influenced by the asymmetry in cortical function perceiving lateral head acceleration.

Associations between functional asymmetry in the otolith reflex pathway and MS susceptibility have been repeatedly demonstrated. For example, Singh et al. (27) reported that bilateral asymmetry in vestibular-evoked myogenic potentials originating from the otolith organs was significantly higher in individuals susceptible to MS than in controls. In addition, it is well documented that astronauts with higher OCR asymmetry were more prone to space MS (8, 11–16). Such accumulated evidence including ours is consistent with the most accepted theory regarding the etiology of MS, the so-called sensory mismatch theory (7, 8), although it suggests that sensory mismatch not only between different sensory modalities but also within the vestibular sense is capable of causing MS.

The current study was conducted under the assumption that OCR asymmetry represents functional asymmetry in the bilateral otolith organs, particularly the utricle. This assumption stems from the fact that the utricle is more sensitive to outward (ipsilateral) rather than inward (contralateral) head-tilt (43). Although the OCR is always driven by both left and right utricular signals (44), such a directional bias in the utricular sensitivity results in a right utricle dominance for the OCR with a rightward head tilt and vice versa. However, we cannot unequivocally rule out another source of OCR asymmetry. That is, the functional asymmetry in the an efferent, as well as afferent, pathway for the OCR, can also cause OCR asymmetry. For example, imbalanced contraction characteristics of extraocular muscles (i.e., the superior and inferior oblique muscles), if any, result in OCR asymmetry. Although the mechanisms by which functional asymmetries in the efferent pathway are associated with MS susceptibility are unclear, identifying the source of OCR asymmetry is an important research direction to further understand the pathogenesis of MS.

Contrasting with the OCR asymmetry, functional asymmetry in a vestibular cortical pathway, assessed by the HT-SVV test, was not associated with MS susceptibility. This may be explained by the central compensation for vestibular sensation (45). Head motion information detected in the bilateral vestibular organs is integrated into the cortical vestibular system (19). When the information from either left or right otolith is unreliable due to unilateral dysfunction of the inner ear, the cortical vestibular system compensates the unreliable otolith afferent signals using head motion information from visual (46) and/or somatosensory (47) systems. This compensatory mechanism could minimize the impact of bilateral asymmetry in the otolith afferent signals in the vestibular cortical pathway. HT-SVV asymmetry was significantly smaller than OCR asymmetry. Also in the reflex pathway, a lot of studies of unilateral vestibular lesions show that asymmetry in the bilateral vestibulo-ocular reflex is known to be diminished over the long term (45, 48, 49). However, in some studies, such compensation in the otolith reflex pathway is incomplete as compared to that in the cortical pathway (45, 50). Our findings are consistent with this evidence. The difference between the patients with unilateral vestibular disorders and the participants in this study is that the participants did not show any difficulties in their daily life or any atypical vestibular functions according to self-reports and screening tests. The reason why the MS-susceptible participants had no difficulties in their daily activities may be because asymmetry in their vestibular function had been corrected, probably by a mechanism similar to the compensatory function in patients. Nevertheless, the MS-susceptible participants had problems specifically in vehicles. This fact may suggest that acceleration during vehicle rides differs from that in other daily activities, such that influences in bilateral vestibular asymmetry appears during vehicle rides, but not during low-motion daily activities.

It should be noted, however, that OCR asymmetry is not the only factor that determines MS susceptibility. Our data showed that a certain portion of participants susceptible to MS was comparable with the control group in terms of OCR asymmetry. This fact enables us to infer the existence of other dominant factors for MS susceptibility, independent of OCR asymmetry. According to our additional analysis for the MSSQ subscale scores, individuals susceptible to MS with lower OCR asymmetry tended to be more prone to MS in swings and roundabouts. Although the peculiarities of motion stimuli in swings and roundabouts compared to other vehicles are unknown, acceleration environments in such playground equipment could reveal other vestibular functions that cannot be accessed using the OCR test. A potential candidate could be the saccular function since the OCR is considered to reflect mainly utricular responses to interaural acceleration accompanied with lateral head tilt (51, 52). In fact, Singh et al. (27) reported that individual MS susceptibility was associated not only with utricular functional asymmetry but also with saccular functional asymmetry, using vestibular evoked potentials. To identify individuals who are prone to MS more accurately, discovering complementary factors for OCR asymmetry is an important future research direction.

This study has certain limitations. A major limitation is the small sample size. This resulted from the high exclusion rate of participants during the OCR measurement. Follow-up studies with a larger sample size are warranted to confirm our findings. To achieve that, a more robust measurement system and algorithm for the OCR are desired. Another limitation is the use of OCR to assess otolith function; in general, the OCR is measured in the photopic condition. In this study, we, thus, followed general methods; however, the OCR in the photopic condition could be compromised by the torsional optokinetic reflex because rotational retinal slips occur accompanying lateral head tilts. In addition, the OCR is primarily associated with utricular function. It is an important future research direction to assess vestibular functions that associate with MS susceptibility using VEMP because VEMP and OCR are complemental for vestibular assessment (36). In addition, as several studies suggested, the role of individual differences in other cortical functions, such as multisensory integration and psychological processes, in the development of MS should also be considered.

### 4.1. Conclusions

Accumulating evidence suggests that individual variations in vestibular functions are associated with MS susceptibility. In the current study, we further investigated whether vestibular functions in the reflex and cortical pathways may be associated with individual MS susceptibility, using a two-group comparison method. This was the first attempt to measure vestibular functions in both the reflex and cortical pathways in the same study population from the perspective of MS susceptibility. Consequently, we found that functional asymmetry in bilateral otoliths in the reflex rather than in the vestibular-cortical pathway is capable of predicting individuals susceptible to MS to some extent. However, our data also suggest that the association between vestibular function and MS susceptibility can vary based on the motion environments provided by vehicles. Future vehicles, such as self-driving cars, could permit a variety of seat layouts. The accompanying diversification of motion environments could make us aware of unexpected vestibular functions associated with MS susceptibility.

## Data Availability Statement

The datasets presented in this article are not readily available because of privacy restrictions in the written informed consent provided by the participants that prevent them from being shared with third parties. Requests to access the datasets should be directed to Tomoko Sugawara, sugawara@mosk.tytlabs.co.jp.

## Ethics Statement

The studies involving human participants were reviewed and approved by the Institutional Review Board of Toyota Central Research & Development Laboratories, Inc. and the Ethics Committee of Nara Medical University. The patients/participants provided their written informed consent to participate in this study.

## Author Contributions

TS, HS, and YW designed the study. TS built the processing program for torsional estimation and analyzed data. TS and HS wrote the manuscript. All the authors were involved in the execution of the experiment and approved the manuscript.

## Funding

This study received funding from Toyota Central Research & Development Laboratories, Inc. The funder had no role in the study design, data collection and analysis, decision to publish, or preparation of the manuscript.

## Conflict of Interest

TS and HS were employed by Toyota Central Research & Development Laboratories, Inc. The remaining authors declare that the research was conducted in the absence of any commercial or financial relationships that could be construed as a potential conflict of interest.

## Publisher's Note

All claims expressed in this article are solely those of the authors and do not necessarily represent those of their affiliated organizations, or those of the publisher, the editors and the reviewers. Any product that may be evaluated in this article, or claim that may be made by its manufacturer, is not guaranteed or endorsed by the publisher.
