# Supplementary material for: Bilateral Asymmetry in Ocular Counter-Rolling Reflex Is Associated With Individual Motion Sickness Susceptibility
Source: Front Neurol. 2021 Nov 19;12:759764. doi: 10.3389/fneur.2021.759764 (PMC8640245; doi:10.3389/fneur.2021.759764)
Supplement: Supplementary file 1 [file Data_Sheet_1.PDF]

Supplementary information

1 VESTIBULAR TESTS FOR SCREENING

1.1 Body sway test

Body sway test was conducted by experimenters using a posturography system (Anima G-5500, Tokyo, Japan). Participants were asked to stand quietly on a rubber mat laid on the posturography plate with closed heels, open toes by 30° each other, fixating on a visual cue (a red circle with a 1 cm diameter on the wall 2 m ahead at eye-level). The system recorded posturography with open eyes for 1 minute. Following that, participants were asked to close their eyes and measured posturography for 1 minute with closed eyes. Software of the device automatically calculated trajectory, area and deviations of trajectories along fore-aft and lateral axes (sway X and sway Y in Table S1, respectively), adding to Romberg ratio (eye-closed/eye-open ratio of trajectory and area) from postulography.

1.2 vHIT

The vHIT was performed by an otolaryngologist in a lighted room. Participants sat on a stool and wore a head-mounted video oculography (VOG) device (EyeSeeCam, EyeSeeTec GmbH, Germany). Participants were asked to fixate on a visual cue (a red circle with a 1 cm wide diameter fixed on the wall at eye-level) with natural blinking throughout the rest. The experimenter held the participant's head and rotated the head in its horizontal plane with impulsive velocity (with maximal velocity >150°/s) by 20° or 30° from the original position. After the head returned to the original position slowly, the same procedure was repeated 15 or more times each in the left and right directions. During the entire procedure, the VOG device recorded the participant's left eye image and head angular velocity with a sampling rate of 220 Hz. The software of the VOG device automatically calculated the left and right HIT gains, H_l and H_r, respectively, by dividing the eye angular velocity by the head angular velocity at 60 ms from the onset of the head rotation.

Table S1. Mean and standard error of mean (SEM) of vestibular function tests in motion sickness susceptible (MSS) and control groups. Results of body sway test are shown from eye-closed (EC) session excepting Romberg ratio.

	MSS ($n = 36$)	Control ($n = 36$)	Effect size g	P value
Trajectory EC [cm]	171±10.5	175±12.3	0.07	0.83
Romberg ratio of trajectory	1.60±0.059	1.58±0.082	0.06	0.89
Area EC [cm^2]	11.2±1.1	11.5±1.3	0.05	0.86
Romberg ratio of area	1.77±0.13	1.68±0.17	0.13	0.70
Sway X EC [cm]	-0.328±0.15	-0.280±0.16	0.07	0.83
Sway Y EC [cm]	-1.29±0.31	-1.49±0.23	0.15	0.60
H_l	1.02 ± 0.030	1.06 ± 0.023	0.22	0.36
H_r	1.12 ± 0.032	1.11 ± 0.031	0.05	0.82
head-upright SVV [°]	1.01± 0.78	1.20 ± 0.74	0.26	0.73

1.3 Head-upright SVV

Head-upright SVV, an index of vestibular function, was culculated as mean deviation of vertical perception from actual vertical both of them were measured in the head-upright posture (see Subsection 3.2 HT-SVV test of MATERIAL AND METHODS in the main text).

2 ESTIMATION OF EYE TORSION

A modified iris template-matching algorithm was used to estimate the eye torsional angle. First, a reference pattern was determined from a video frame of the participant's eye in upright head posture in the ocular counter-rolling (OCR) test as follows: a video frame showing a clear iris pattern was chosen (Figure S1A), and a partial, distinctive iris pattern was extracted from the frame around the pupil, transformed to a rectangular shape, and stored as the reference pattern. Moreover, the position of the reference pattern on the entire pupil circumference was stored as null position. Second, eye torsion in another video frame was estimated as follows: a wider iris area than the reference pattern was extracted from another video frame (Figure S1B) and transformed into a rectangular shape for pattern matching. The transforming algorithm included the algorithm for correcting iris-image distortion using a three-dimensional eyeball rotation model, aqueous humor refraction model (Japanese Patent: JP6503856B2), and iris elasticity model (Nishiyama et al., 2008) accompanied with changes of pupil diameter. The stored reference pattern was searched for the most plausible position in the region for pattern matching (Figure S1C). Then the eye torsional angle at the frame was calculated as the ratio of the discrepancy between the matched and null position to the entire pupil circumference. The same procedure was applied for static, consecutive eye images of 5 s time period excluding blinking, and the mean eye torsional angle of this 5 s part was calculated (Figure 3 in the main text). Differences between the mean eye torsional angle in tilted head posture and in upright head posture just before the tilting period were used as the estimated eye torsional angles for calculating the OCR gains.

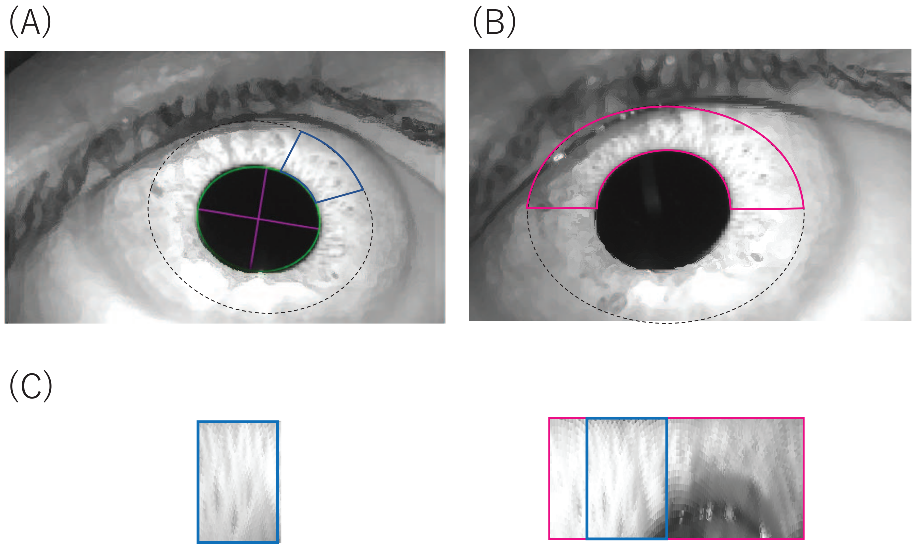

Figure S1. (A): A processed eye image and the reference iris pattern (bold line) in the upright head position. (B): Region of pattern matching (bold line) from the same individual as (A) during head tilt. (C): The reference pattern in the transformed shape (left) from (A) and its plausible position (narrow rectangle in right figure) in the transformed region of the pattern matching (wide rectangle in right figure) from (B).

REFERENCES

Nishiyama, J., Hashimoto, T., Sakashita, Y., Fujiyoshi, H., and Hirata, Y. (2008). Characterization of iris pattern stretches and application to the measurement of roll axis eye movements. In *2008 30th Annual International Conference of the IEEE Engineering in Medicine and Biology Society* (IEEE), 4949–4952
